# 4-Chloro-6-methyl-*N*-(4-methyl­phen­yl)quinolin-2-amine

**DOI:** 10.1107/S1600536811002327

**Published:** 2011-01-26

**Authors:** K. N. Vennila, K. Prabha, M. Manoj, K. J. Rajendra Prasad, D. Velmurugan

**Affiliations:** a Centre of Advanced Study in Crystallography and Biophysics, University of Madras, Guindy Campus, Chennai 600 025, India; b Department of Chemistry, Bharathiar University, Coimbatore 641 046, India

## Abstract

In the title compound C_17_H_15_ClN_2_, the dihedral angle between the quinoline ring system and the phenyl ring is 50.18 (6)°. In the crystal, mol­ecules are linked into chains running along the *c* axis by N—H⋯N hydrogen bonds.

## Related literature

For the biological activity of quinoline derivatives, see: Lunniss *et al.* (2009 ▶); Kemnitzer *et al.* (2008 ▶); Woodrow *et al.* (2009 ▶). For a related structure, see: Cheng *et al.* (2005 ▶). For the synthesis, see: Manoj *et al.* (2011 ▶).
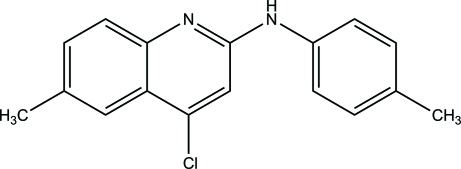


## Experimental

### 

#### Crystal data

C_17_H_15_ClN_2_
*M* *_r_* = 282.76
Monoclinic, 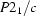

*a* = 15.1445 (13) Å
*b* = 11.4337 (10) Å
*c* = 8.4764 (7) Å
β = 92.344 (4)°
*V* = 1466.5 (2) Å^3^
*Z* = 4
Mo *K*α radiation
μ = 0.25 mm^−1^
*T* = 293 K
0.22 × 0.21 × 0.20 mm

#### Data collection

Bruker SMART APEXII CCD diffractometer
13649 measured reflections
3669 independent reflections
2508 reflections with *I* > 2σ(*I*)
*R*_int_ = 0.027

#### Refinement

*R*[*F* ^2^ > 2σ(*F* ^2^)] = 0.044
*wR*(*F* ^2^) = 0.125
*S* = 1.03
3669 reflections
183 parameters
H-atom parameters constrained
Δρ_max_ = 0.18 e Å^−3^
Δρ_min_ = −0.30 e Å^−3^

### 

Data collection: *APEX2* (Bruker, 2008 ▶); cell refinement: *SAINT* (Bruker, 2008 ▶); data reduction: *SAINT*; program(s) used to solve structure: *SHELXS97* (Sheldrick, 2008 ▶); program(s) used to refine structure: *SHELXL97* (Sheldrick, 2008 ▶); molecular graphics: *ORTEP-3* (Farrugia, 1997 ▶); software used to prepare material for publication: *PLATON* (Spek, 2009 ▶).

## Supplementary Material

Crystal structure: contains datablocks global, I. DOI: 10.1107/S1600536811002327/bt5456sup1.cif

Structure factors: contains datablocks I. DOI: 10.1107/S1600536811002327/bt5456Isup2.hkl

Additional supplementary materials:  crystallographic information; 3D view; checkCIF report

## Figures and Tables

**Table 1:** Hydrogen-bond geometry (Å, °)

*D*—H⋯*A*	*D*—H	H⋯*A*	*D*⋯*A*	*D*—H⋯*A*
N2—H2⋯N1^i^	0.86	2.59	3.404 (2)	157

Symmetry code: (i) 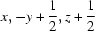.

**Fig. 1.**
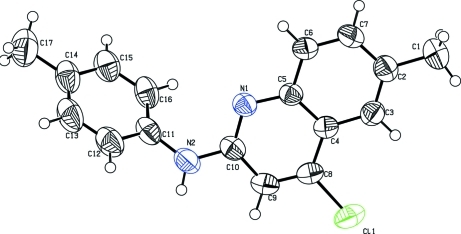
View of the title molecule, showing the thermal ellipsoids drawn at the 50% probability level.

**Fig. 2.**
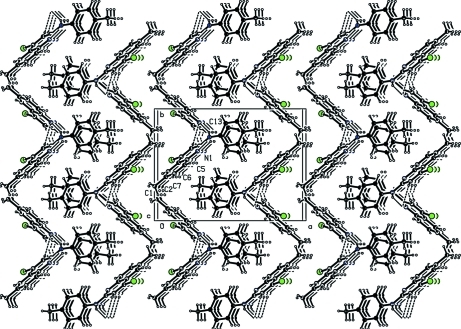
The crystal packing of the title compound viewed down c-axis with bifurcated hydrogen bonds (dotted lines) between the molecules.

## supplementary crystallographic information

### Comment

Quinoline derivatives fused with various heterocycles have displayed potent anticancer activity targeting different sites like topoisomerase I, telomerase, farnasyl transferase, Src tyrosine kinase, protein kinase CK-II etc. The trisubstituted quinoline analogs have attractive profile and are a good start point to initiate a lead optimisation programme. Due to its significant biological importance, the title compound was chosen for the X-ray crystallographic study.

The title compound is the first structural example with methyl phenyl moiety attached to the amino substituted quinoline. The chlorine atom deviates only by 0.0276 (5)Å below the mean plane of atoms passing through C2-C10, N1. The methyl phenyl moiety is oriented at 129.9 (5)° from the plane containing the quinoline ring system.

C-H···N and N-H···N intermolecular interactions assist the molecular packing of the crystal which resembles helical patterns. In addition to van der Waals forces,  the hydrogen bond interactions at the groove of helix maintains the stability of the crystal packing. Atom N1 acts as a bifurcated acceptor. The bifurcated hydrogen bond also forms the R_1_^2^(6) motif. The structure of the title compound is shown in Figure 1. All the bond lengths and bond angles are in the usual ranges. The molecular packing with hydrogen bonds as dotted lines is shown in Figure 2.

### Experimental

A mixture of appropriate 6-methyl-2,4-dichloroquinoline (0.010 mol) and *p*-toluidine (0.010 mol) was heated under neat condition at 160°C for half an hour. The product obtained was washed with water, dried and purified by column chromatography over silica gel and eluted with petroleum ether : ethyl acetate mixture (99 : 1) to get the product as pale yellow solid. It was recrystallised using methanol.

### Refinement

The H-atoms were positioned geometrically and treated as riding atoms: C—H =0.93 Å H-aromatic, C—H = 0.96 Å H-methyl, and N—H = 0.86 Å, with *U*_iso_ = k×*U*_eq_(parent C or N-atom), where k = 1.5 for methyl H-atoms, and = 1.2 for all other H-atoms.

### Crystal data

**Table:**

C_17_H_15_ClN_2_	*F*(000) = 592
*M**_r_* = 282.76	*D*_x_ = 1.281 Mg m^−^^3^
Monoclinic, *P*2_1_/*c*	Mo *K*α radiation, λ = 0.71073 Å
Hall symbol: -P 2ybc	Cell parameters from 3683 reflections
*a* = 15.1445 (13) Å	θ = 1.3–28.4°
*b* = 11.4337 (10) Å	µ = 0.25 mm^−^^1^
*c* = 8.4764 (7) Å	*T* = 293 K
β = 92.344 (4)°	Block, yellow
*V* = 1466.5 (2) Å^3^	0.22 × 0.21 × 0.20 mm
*Z* = 4	

### Data collection

**Table:**

Bruker SMART APEXII CCD diffractometer	2508 reflections with *I* > 2σ(*I*)
Radiation source: fine-focus sealed tube	*R*_int_ = 0.027
graphite	θ_max_ = 28.4°, θ_min_ = 1.4°
ω and φ scans	*h* = −17→20
13649 measured reflections	*k* = −13→15
3669 independent reflections	*l* = −11→11

### Refinement

**Table:**

Refinement on *F*^2^	Primary atom site location: structure-invariant direct methods
Least-squares matrix: full	Secondary atom site location: difference Fourier map
*R*[*F*^2^ > 2σ(*F*^2^)] = 0.044	Hydrogen site location: inferred from neighbouring sites
*wR*(*F*^2^) = 0.125	H-atom parameters constrained
*S* = 1.03	*w* = 1/[σ^2^(*F*_o_^2^) + (0.052*P*)^2^ + 0.3324*P*] where *P* = (*F*_o_^2^ + 2*F*_c_^2^)/3
3669 reflections	(Δ/σ)_max_ = 0.001
183 parameters	Δρ_max_ = 0.18 e Å^−^^3^
0 restraints	Δρ_min_ = −0.30 e Å^−^^3^

### Special details

**Table:**

Geometry. All esds (except the esd in the dihedral angle between two l.s. planes) are estimated using the full covariance matrix. The cell esds are taken into account individually in the estimation of esds in distances, angles and torsion angles; correlations between esds in cell parameters are only used when they are defined by crystal symmetry. An approximate (isotropic) treatment of cell esds is used for estimating esds involving l.s. planes.
Refinement. Refinement of F^2^ against ALL reflections. The weighted R-factor wR and goodness of fit S are based on F^2^, conventional R-factors R are based on F, with F set to zero for negative F^2^. The threshold expression of F^2^ > 2sigma(F^2^) is used only for calculating R-factors(gt) etc. and is not relevant to the choice of reflections for refinement. R-factors based on F^2^ are statistically about twice as large as those based on F, and R- factors based on ALL data will be even larger.

### Fractional atomic coordinates and isotropic or equivalent isotropic displacement parameters (Å^2^)

**Table:**

	*x*	*y*	*z*	*U*_iso_*/*U*_eq_	
Cl1	0.88432 (3)	0.45826 (5)	0.04769 (5)	0.07158 (19)	
C4	0.83107 (10)	0.48222 (13)	−0.26320 (17)	0.0457 (4)	
N1	0.70049 (9)	0.37817 (12)	−0.37362 (14)	0.0493 (3)	
C5	0.77053 (10)	0.45280 (13)	−0.38898 (17)	0.0452 (3)	
C6	0.78424 (12)	0.50298 (17)	−0.53791 (19)	0.0571 (4)	
H6	0.7459	0.4847	−0.6228	0.068*	
C3	0.90078 (11)	0.55972 (15)	−0.2897 (2)	0.0537 (4)	
H3	0.9400	0.5786	−0.2063	0.064*	
C8	0.81569 (11)	0.42723 (14)	−0.11597 (17)	0.0490 (4)	
C9	0.74846 (12)	0.35319 (15)	−0.10002 (19)	0.0545 (4)	
H9	0.7394	0.3172	−0.0036	0.065*	
N2	0.62355 (11)	0.25326 (14)	−0.21237 (17)	0.0660 (4)	
H2	0.6281	0.2093	−0.1301	0.079*	
C7	0.85260 (12)	0.57759 (16)	−0.5596 (2)	0.0610 (5)	
H7	0.8599	0.6092	−0.6593	0.073*	
C2	0.91267 (12)	0.60825 (16)	−0.4350 (2)	0.0578 (4)	
C11	0.54756 (13)	0.23837 (16)	−0.3114 (2)	0.0585 (4)	
C10	0.69089 (11)	0.33048 (14)	−0.23354 (19)	0.0509 (4)	
C16	0.50425 (12)	0.33144 (16)	−0.3851 (2)	0.0633 (5)	
H16	0.5279	0.4063	−0.3755	0.076*	
C14	0.38882 (13)	0.2042 (2)	−0.4889 (2)	0.0721 (6)	
C15	0.42658 (13)	0.31429 (18)	−0.4723 (2)	0.0691 (5)	
H15	0.3988	0.3780	−0.5213	0.083*	
C12	0.51110 (15)	0.12859 (18)	−0.3286 (2)	0.0754 (6)	
H12	0.5393	0.0646	−0.2812	0.091*	
C1	0.98772 (14)	0.69163 (19)	−0.4625 (3)	0.0794 (6)	
H1A	0.9647	0.7693	−0.4775	0.119*	
H1B	1.0178	0.6680	−0.5549	0.119*	
H1C	1.0284	0.6907	−0.3727	0.119*	
C17	0.30336 (15)	0.1871 (2)	−0.5837 (3)	0.1002 (8)	
H17A	0.3034	0.1115	−0.6330	0.150*	
H17B	0.2977	0.2465	−0.6635	0.150*	
H17C	0.2546	0.1923	−0.5152	0.150*	
C13	0.43288 (16)	0.1126 (2)	−0.4157 (3)	0.0818 (7)	
H13	0.4093	0.0377	−0.4251	0.098*	

### Atomic displacement parameters (Å^2^)

**Table:**

	*U*^11^	*U*^22^	*U*^33^	*U*^12^	*U*^13^	*U*^23^
Cl1	0.0732 (3)	0.0915 (4)	0.0487 (3)	0.0072 (3)	−0.0138 (2)	0.0015 (2)
C4	0.0506 (8)	0.0431 (8)	0.0435 (8)	0.0052 (7)	0.0014 (6)	−0.0006 (6)
N1	0.0576 (8)	0.0490 (7)	0.0415 (7)	−0.0057 (6)	0.0054 (6)	−0.0029 (6)
C5	0.0513 (8)	0.0439 (8)	0.0406 (7)	−0.0002 (7)	0.0037 (6)	−0.0011 (6)
C6	0.0644 (10)	0.0655 (11)	0.0411 (8)	−0.0082 (9)	−0.0010 (7)	0.0034 (8)
C3	0.0536 (9)	0.0528 (10)	0.0542 (9)	−0.0011 (8)	−0.0037 (7)	−0.0015 (8)
C8	0.0565 (9)	0.0508 (9)	0.0395 (8)	0.0090 (8)	−0.0011 (7)	−0.0007 (7)
C9	0.0697 (11)	0.0531 (10)	0.0410 (8)	0.0055 (9)	0.0069 (7)	0.0060 (7)
N2	0.0813 (10)	0.0610 (9)	0.0561 (9)	−0.0195 (8)	0.0079 (8)	0.0082 (7)
C7	0.0694 (11)	0.0659 (12)	0.0483 (9)	−0.0085 (9)	0.0076 (8)	0.0112 (8)
C2	0.0571 (9)	0.0523 (10)	0.0641 (11)	−0.0037 (8)	0.0049 (8)	0.0028 (8)
C11	0.0720 (11)	0.0512 (10)	0.0540 (9)	−0.0145 (9)	0.0211 (8)	−0.0070 (8)
C10	0.0622 (10)	0.0443 (9)	0.0469 (9)	−0.0012 (8)	0.0106 (7)	−0.0016 (7)
C16	0.0644 (11)	0.0509 (11)	0.0759 (12)	−0.0124 (9)	0.0198 (9)	−0.0141 (9)
C14	0.0716 (12)	0.0801 (14)	0.0660 (12)	−0.0248 (11)	0.0220 (10)	−0.0215 (11)
C15	0.0644 (11)	0.0662 (13)	0.0780 (13)	−0.0061 (9)	0.0198 (10)	−0.0078 (10)
C12	0.0959 (15)	0.0530 (11)	0.0781 (13)	−0.0185 (11)	0.0119 (11)	−0.0002 (10)
C1	0.0747 (13)	0.0750 (14)	0.0886 (15)	−0.0199 (11)	0.0065 (11)	0.0112 (11)
C17	0.0856 (16)	0.121 (2)	0.0946 (17)	−0.0340 (15)	0.0107 (13)	−0.0253 (16)
C13	0.0992 (17)	0.0598 (13)	0.0876 (15)	−0.0334 (12)	0.0173 (13)	−0.0139 (11)

### Geometric parameters (Å, °)

**Table:**

Cl1—C8	1.7356 (16)	C2—C1	1.509 (3)
C4—C3	1.403 (2)	C11—C12	1.377 (3)
C4—C5	1.418 (2)	C11—C16	1.385 (3)
C4—C8	1.425 (2)	C16—C15	1.378 (3)
N1—C10	1.320 (2)	C16—H16	0.9300
N1—C5	1.3716 (19)	C14—C13	1.376 (3)
C5—C6	1.410 (2)	C14—C15	1.387 (3)
C6—C7	1.360 (2)	C14—C17	1.508 (3)
C6—H6	0.9300	C15—H15	0.9300
C3—C2	1.369 (2)	C12—C13	1.382 (3)
C3—H3	0.9300	C12—H12	0.9300
C8—C9	1.335 (2)	C1—H1A	0.9600
C9—C10	1.424 (2)	C1—H1B	0.9600
C9—H9	0.9300	C1—H1C	0.9600
N2—C10	1.366 (2)	C17—H17A	0.9600
N2—C11	1.407 (2)	C17—H17B	0.9600
N2—H2	0.8600	C17—H17C	0.9600
C7—C2	1.410 (2)	C13—H13	0.9300
C7—H7	0.9300		
			
C3—C4—C5	119.79 (14)	N1—C10—N2	119.69 (15)
C3—C4—C8	124.74 (14)	N1—C10—C9	123.58 (15)
C5—C4—C8	115.47 (14)	N2—C10—C9	116.71 (15)
C10—N1—C5	117.16 (13)	C15—C16—C11	120.72 (17)
N1—C5—C6	118.75 (14)	C15—C16—H16	119.6
N1—C5—C4	123.71 (13)	C11—C16—H16	119.6
C6—C5—C4	117.54 (14)	C13—C14—C15	117.0 (2)
C7—C6—C5	121.16 (15)	C13—C14—C17	122.1 (2)
C7—C6—H6	119.4	C15—C14—C17	120.8 (2)
C5—C6—H6	119.4	C16—C15—C14	121.5 (2)
C2—C3—C4	121.83 (15)	C16—C15—H15	119.2
C2—C3—H3	119.1	C14—C15—H15	119.2
C4—C3—H3	119.1	C11—C12—C13	120.5 (2)
C9—C8—C4	121.40 (14)	C11—C12—H12	119.7
C9—C8—Cl1	118.87 (12)	C13—C12—H12	119.7
C4—C8—Cl1	119.72 (13)	C2—C1—H1A	109.5
C8—C9—C10	118.67 (14)	C2—C1—H1B	109.5
C8—C9—H9	120.7	H1A—C1—H1B	109.5
C10—C9—H9	120.7	C2—C1—H1C	109.5
C10—N2—C11	126.62 (15)	H1A—C1—H1C	109.5
C10—N2—H2	116.7	H1B—C1—H1C	109.5
C11—N2—H2	116.7	C14—C17—H17A	109.5
C6—C7—C2	121.75 (16)	C14—C17—H17B	109.5
C6—C7—H7	119.1	H17A—C17—H17B	109.5
C2—C7—H7	119.1	C14—C17—H17C	109.5
C3—C2—C7	117.93 (16)	H17A—C17—H17C	109.5
C3—C2—C1	121.50 (17)	H17B—C17—H17C	109.5
C7—C2—C1	120.56 (17)	C14—C13—C12	121.99 (19)
C12—C11—C16	118.19 (19)	C14—C13—H13	119.0
C12—C11—N2	119.22 (19)	C12—C13—H13	119.0
C16—C11—N2	122.46 (16)		
			
C10—N1—C5—C6	178.35 (15)	C6—C7—C2—C1	−179.77 (18)
C10—N1—C5—C4	−1.1 (2)	C10—N2—C11—C12	146.26 (19)
C3—C4—C5—N1	−179.84 (14)	C10—N2—C11—C16	−38.0 (3)
C8—C4—C5—N1	0.9 (2)	C5—N1—C10—N2	−178.42 (15)
C3—C4—C5—C6	0.7 (2)	C5—N1—C10—C9	0.4 (2)
C8—C4—C5—C6	−178.59 (15)	C11—N2—C10—N1	−17.5 (3)
N1—C5—C6—C7	179.95 (16)	C11—N2—C10—C9	163.54 (16)
C4—C5—C6—C7	−0.5 (3)	C8—C9—C10—N1	0.4 (3)
C5—C4—C3—C2	−0.2 (3)	C8—C9—C10—N2	179.33 (15)
C8—C4—C3—C2	178.95 (16)	C12—C11—C16—C15	0.3 (3)
C3—C4—C8—C9	−179.17 (16)	N2—C11—C16—C15	−175.44 (16)
C5—C4—C8—C9	0.0 (2)	C11—C16—C15—C14	0.4 (3)
C3—C4—C8—Cl1	1.9 (2)	C13—C14—C15—C16	−0.7 (3)
C5—C4—C8—Cl1	−178.91 (11)	C17—C14—C15—C16	179.50 (18)
C4—C8—C9—C10	−0.7 (2)	C16—C11—C12—C13	−0.7 (3)
Cl1—C8—C9—C10	178.30 (12)	N2—C11—C12—C13	175.21 (18)
C5—C6—C7—C2	0.0 (3)	C15—C14—C13—C12	0.3 (3)
C4—C3—C2—C7	−0.4 (3)	C17—C14—C13—C12	−179.9 (2)
C4—C3—C2—C1	179.92 (17)	C11—C12—C13—C14	0.4 (3)
C6—C7—C2—C3	0.5 (3)		

### Hydrogen-bond geometry (Å, °)

**Table:**

*D*—H···*A*	*D*—H	H···*A*	*D*···*A*	*D*—H···*A*
N2—H2···N1^i^	0.86	2.59	3.404 (2)	157

Symmetry codes: (i) *x*, −*y*+1/2, *z*+1/2.
